# Molecular Insights in Psychiatry

**DOI:** 10.3390/ijms23094878

**Published:** 2022-04-28

**Authors:** Giovanna Traina, Jack Adam Tuszynski, Massimo Cocchi

**Affiliations:** 1 Department of Pharmaceutical Sciences, University of Perugia, 06126 Perugia, Italy; 2 Experimental Oncology, University of Alberta, Edmonton, AB T6G 1Z2, Canada; jack.tuszynski@gmail.com; 3 Department of Mechanical and Aerospace Engineering, Politecnico di Torino, Corso Duca degli Abruzzi 24, 10129 Turin, Italy; 4 Department of Veterinary Medical Sciences, University of Bologna, Ozzano dell’Emilia, 40064 Bologna, Italy; massimo.cocchi@unibo.it

This Special Issue included articles discussing several important psychiatric phenomena whose elucidation can be provided by cellular and subcellular molecular mechanisms. Below we provide an overview of the main findings reported in these contributions to the Special Issue.

Chronic stress plays a significant role in the onset of severe and disabling psychiatric conditions. Their onset or the exaggeration of their symptoms could be induced by a wide variety of factors including genetic predispositions, infections, inflammations, alterations of trace elements and vitamins, severe stress, neurodegenerative diseases, trauma, stimulants and psychological factors.

The brain is particularly vulnerable to oxidative stress. Oxidative stress resulting from environmental risk factors associated with neurodevelopmental disorders alters the transcriptional landscape of neurons during differentiation. To test this hypothesis, Ref. [1] used the most widely accepted in vitro model for neurodevelopmental and neuropsychiatric studies, the SH-SY5Y neuroblastoma cell line. The authors induced the differentiation of neuroblasts in the presence of oxidative stress and examined gene expression in mature neuronal cells. A differential gene expression of thousands of genes has emerged, which revealed those genes and pathways which are largely involved in the development of neuronal and cardiovascular systems as well as in processes related to immunity. Specifically, signaling pathways associated with schizophrenia have been identified. Oxidative stress is a key risk factor in the onset of neurodevelopmental disorders, potentially through disruption of neurogenesis and differentiation, as well as dysregulation of immunomodulatory genes in neuronal progenitor cells. Various classes of functional genes in the immune system, including the complement family, cytokines, chemokines, and Toll-like receptors, exhibit pleiotropy with functions related to neural development. Oxidative stress is likely to have adverse effects even before the onset of neuronal differentiation, suggesting that neuronal progenitor cells in the fetal nervous system may be particularly vulnerable to this exposure in the very early period of pregnancy, posing a risk of developing psychiatric disorders during the person’s lifetime.

The review by Baj et al. [2] focuses on the relationship between the levels of various elements, including chromium, copper, iron, manganese, selenium, or zinc, and schizophrenia. These elements are crucial in maintaining the proper functioning of the central nervous system, and changes in their levels can induce CNS disease or exacerbate existing symptoms of disorders, such as neurodegenerative disorders, dementia, or major depressive disorder. Various studies have shown altered levels of serum concentrations of trace elements in schizophrenic patients. Among the numerous triggers, nutritional deprivation has a significant impact, inducing oxidative stress, irrational behavior and cognitive disturbances. Furthermore, an association was highlighted between exposure to metals with consequent imbalances in concentration and severity of psychotic symptoms. However, the results of the studies analyzed were generally contradictory and the epidemiological evidence of altered serum levels of trace elements and the risk of developing schizophrenia is somewhat controversial. The differences in the results of these studies may be due to various methodological criticalities, the heterogeneity of the methodology, as well as the different inclusion and exclusion criteria. Furthermore, the concentrations of trace elements are usually determined only in the extracellular compartments, while some of the elements (e.g., Mg) are specifically intracellular ions. Finally, there are no strict normal concentration ranges for most elements. According to the results of the analyzed studies, maternal deficiency of essential trace elements with micro and macronutrients together with the subsequent dysregulation of trace element concentrations in early childhood could be associated with an increased risk of psychotic disorders including schizophrenia.

In their article, Ref. [3] reported the results of their studies of the effects that sound, pressure waves and mechanical vibrations have on cells and their components. Starting from the evidence that cells are able to communicate with each other through light emissions and in general through electromagnetic waves, the authors hypothesize that cells can also communicate through mechanical vibrations. Acoustic stimuli are important in guiding the spatial interaction between cells, influencing their individual and collective behavior, as well as their intracellular and intercellular organization.

The microtubules of the cytoskeleton, made up of heterodimers of alpha and beta tubulin as their building blocks, interact with the other proteins of the cytoskeleton and are responsible for the structure and shape of the cell and its movements including their critical role in cell division as components of mitotic spindles. They also interact with other cytoplasmic organelles, such as mitochondria, and regulate their functions.

Ref. [3] analyzed how information on wavelength and frequency encoded in sounds and phonemes, as well as the same words used by the subjects in therapy, could influence heart function. It has been observed that the contractility and spatial organization of HL1 cardiac cells are influenced by sound stimuli that produce a different localization and fluorescence emission of cytoskeletal proteins. It emerged that in response to meditative practice, listening to meditative music, the activity of mantras and expressions of love, the intensity of the emission of light by phalloidin (F-actin), the beta-actin and alpha-actinin-1 (which builds bridges between F-actin filaments) increased, while after expressions of hatred a decrease in emissions of the same markers was observed. This could reflect a change in molecular polymerization that could reveal the change in cell contractility. The authors propose a mechanistic physics-based model to explain their results, based precisely on coherent molecular dynamics. The fractal and multifractal structure of the sound used and the cellular response suggest that the coherence of the underlying molecular dynamics plays an important role in the phenomena studied by these authors.

Schizophrenia is a chronic and debilitating disease accompanied by impaired social functions. A review by Ref. [4] analyzes how oxytocin dysregulation may play a role in the regulation of schizophrenia expression. Oxytocin is known to play an important role in developing attachment between children and parents through early contact and interaction. Evidence supports the idea that oxytocin could facilitate social interaction and modulate non-verbal interpersonal communication. The potential clinical benefits of oxytocin in improving psychopathology in patients with schizophrenia were examined. Lower levels of endogenous oxytocin are associated with more severe positive symptoms in patients with schizophrenia. Intranasal oxytocin application increases the activity of the amygdala, hippocampus, parahippocampal gyrus and putamen and increases communication between the amygdala, insula and caudate. However, the involvement of oxytocin in social bonding in humans shows contextual and interindividual variability. Treatment with oxytocin significantly modulates social perception by reducing attention to negative facial expressions, and the best results were seen with oxytocin treatment when social feedback was provided. Studies have shed light on the therapeutic potential that oxytocin has in patients with schizophrenia whose social cognition is impaired.

Ref. [5] summarized the role of smoking in neuropsychiatric diseases. Smoking is known to be associated with an increased incidence of lung cancer and respiratory disease, however modern analytical methods and large clinical trials have expanded these findings. Exposure to smoke is chronic, and the toxic molecules that accumulate can affect a whole spectrum of diseases. It emerged that the high quantity of toxic compounds produced by the combustion of tobacco cigarettes has a significant role in the onset and progression of many diseases, such as cardiovascular and neurovascular diseases, as well as metabolic and neuropsychiatric diseases. Toxic chemical groups present in tobacco smoke are labeled carcinogens, such as tobacco-specific nitrosamines and polycyclic aromatic hydrocarbons. On the other hand, volatile organic compounds, which are able to cross the blood–brain barrier, and carbonyl compounds, which can generate protein adducts, can be implicated in many other different diseases.
